# Parkinson’s disease: proteinopathy or lipidopathy?

**DOI:** 10.1038/s41531-019-0103-7

**Published:** 2020-01-03

**Authors:** Saranna Fanning, Dennis Selkoe, Ulf Dettmer

**Affiliations:** Ann Romney Center for Neurologic Diseases, Department of Neurology, Brigham and Women’s Hospital and Harvard Medical School, Boston, MA 02115 USA

**Keywords:** Parkinson's disease, Cellular neuroscience

## Abstract

Lipids play a more significant role in Parkinson’s disease and its related brain disorders than is currently recognized, supporting a “lipid cascade”. The 14 kDa protein α-synuclein (αS) is strongly associated with Parkinson’s disease (PD), dementia with Lewy bodies (DLB), other synucleinopathies such as multiple system atrophy, and even certain forms of Alzheimer’s disease. Rigorously deciphering the biochemistry of αS in native systems is the key to developing treatments. αS is highly expressed in the brain, the second most lipid-rich organ, and has been proposed to be a lipid-binding protein that physiologically interacts with phospholipids and fatty acids (FAs). αS-rich cytoplasmic inclusions called Lewy bodies and Lewy neurites are the hallmark lesions of synucleinopathies. Excess αS–membrane interactions may trigger proteinaceous αS aggregation by stimulating its primary nucleation. However, αS may also exert its toxicity prior to or independent of its self-aggregation, e.g., via excessive membrane interactions, which may be promoted by certain lipids and FAs. A complex αS-lipid landscape exists, which comprises both physiological and pathological states of αS. As novel insights about the composition of Lewy lesions occur, new lipid-related PD drug candidates emerge, and genome-wide association studies (GWAS) increasingly validate new hits in lipid-associated pathways, it seems timely to review our current knowledge of lipids in PD and consider the roles for these pathways in synucleinopathies.

**Fig. 1:**
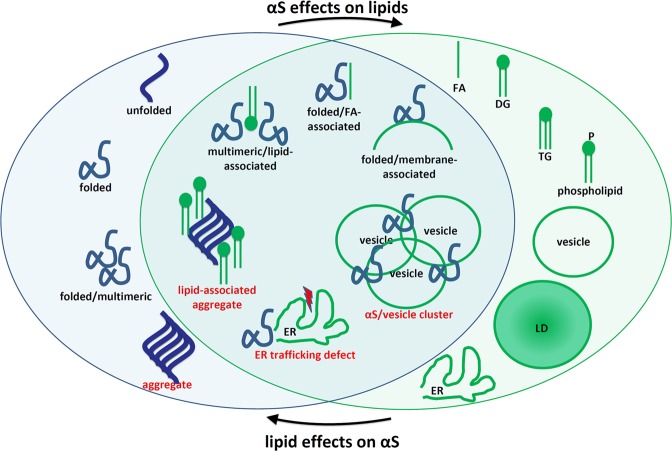
αS ↔ lipid interplay: aspects of cellular αS homeostasis (blue oval), aspects of lipid homeostasis (green oval), and overlapping aspects. Pathological states are labeled in red. Simplified schematic of both select αS and select lipid species. Several existing publications suggest αS effects on lipids and vice versa, as indicated by arrows. DG diglyceride, ER endoplasmic reticulum, FA fatty acid, LD, lipid droplet, TG triglyceride.

Pathological states are labeled in red. Simplified schematic of both select αS and select lipid species. Several existing publications suggest αS effects on lipids and vice versa, as indicated by arrows. DG diglyceride, ER endoplasmic reticulum, FA fatty acid, LD, lipid droplet, TG triglyceride.

## Introduction

The 14 kDa protein α-synuclein (αS) is strongly associated with Parkinson’s disease (PD), dementia with Lewy bodies (DLB), other synucleinopathies such as multiple system atrophy, and even certain forms of Alzheimer’s disease. Rigorously deciphering the biochemistry of αS in native systems is the key to developing treatments. αS is highly expressed in brain, the second most lipid-rich organ,^1^ and has been proposed to be a lipid-binding protein that physiologically interacts with phospholipids^2–5^ and fatty acids (FAs).^6–9^ αS-rich cytoplasmic inclusions called Lewy bodies (LBs) and Lewy neurites are the hallmark lesions of synucleinopathies. Excess αS–membrane interactions may trigger proteinaceous αS aggregation by stimulating its primary nucleation.^10^ However, αS may also exert its toxicity prior to or independent of its self-aggregation, e.g., via excessive membrane interactions,^11,12^ which may be promoted by certain lipids and FAs. A complex αS-lipid landscape exists that comprises both physiological and pathological states of αS (Fig. 1). As novel insights about the composition of Lewy lesions occur,^13^ new lipid-related PD drug candidates emerge^14–16^ and genome-wide association studies (GWAS) increasingly validate new hits in lipid-associated pathways,^17–28^ it seems timely to review our current knowledge of lipids in PD and consider the roles for these pathways in synucleinopathies.

## αS transiently binds to lipid membranes physiologically

**Fig. 2:**
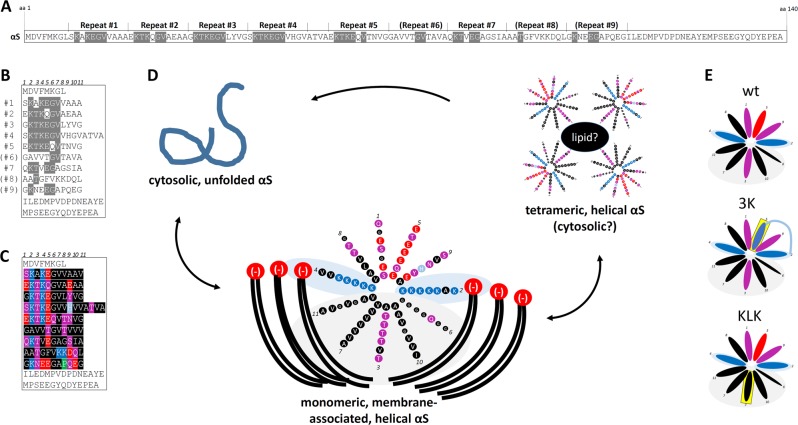
Electrostatic and hydrophobic interactions govern transient αS membrane binding. **a** Amino acid sequence of human wt αS; residues that fully conform to the core repeat motif “KTKEGV” are highlighted in gray. **b** Amino acid sequence of human wt αS displayed by aligning the KTKEGV motifs. **c** Analogous to **b**, color-coded residues: black = uncharged, red = negatively charged, dark blue = positively charged, light blue = histidine, and purple = uncharged and polar. **d** Color-coded schematic of repeats 1–7 (omitting “ATVA” between repeats 4 and 5) in an 11/3 helical wheel, embedded in the outer leaflet of a lipid membrane. **e** Simplified schematics of membrane-induced αS helices: αS wt as well as 3 K (amplified E46K) and KLK (engineered highly hydrophobic). Top: wt. Middle: a proposed increased electrostatic interaction between excess positive charges of the lysines (highlighted in yellow) and phospholipid head groups is indicated by a blue line. Bottom: the KLK variant is stabilized by excess hydrophobicity in the hydrophobic half of the amphipathic helix, highlighted in yellow.

Early biochemical characterization identified αS as soluble^29,30^ and brain extract fractionation showed only a weak association with synaptic vesicles,^31,32^ confirming immunogold-EM that had detected αS throughout cytoplasmic matrices in axon terminals.^33^ Photobleaching microscopy also indicated that αS interacts only weakly with membranes of the nerve terminal and switches rapidly between the cytosol and membrane.^32,34^ The earliest characterizations of αS already suggested that binding of αS to membranes may occur via the formation of amphipathic helices mediated by an 11-amino acid repeat motif having the core consensus sequence KTKEGV.^35^ This motif appears imperfectly six to nine times in the first two-thirds of the protein^35^ (Fig. 2a–c) and resembles lipid-binding domains often observed in apolipoproteins.^29^ “Cis” and “trans” factors mediate the transient αS–membrane interaction when the N-terminal two-thirds of αS form an 11/3 helix (11 amino acids/three turns)^36^ (Fig. 2d). Nonpolar amino acid residues in the hydrophobic half of the αS amphipathic helix “dip” into the membrane bilayer (~1–5 Å below lipid head groups),^37–40^ interacting with the lipid “tails” via van der Waals forces (Fig. 2d). Lysine residues (+) in the KTKEGV motifs interact with membrane lipid head groups (−).^41^ On the “trans” side, vesicle membrane composition^42–46^ and vesicle size affect αS binding: negatively charged head groups promote the interaction with lysine residues (see above) and small vesicles that exhibit “lipid packing defects” promote αS “insertion” into the membrane.^47,48^ The helix formation is only transient, not stable, because some nonpolar residues are found in the hydrophilic half of the helix and some polar residues interact with lipid bilayer, most importantly threonine residues (see Fig. 2d). This imperfect helix formation seems to underlie the transient nature of αS–membrane binding.^49–51^ A recent, elegant in vitro study suggests αS that comes off the membrane does not immediately lose its fold, but may actually retain it, and this may go hand-in-hand with native αS–αS assembly.^45^ The authors studied αS binding to small unilamellar vesicles composed of phosphatidylcholine (PC) with 13:0 fatty acyl side chains. Modulating αS binding via phase transitions of the vesicle lipids allowed the reconstitution of soluble helical αS species that behaved as multimers. These observations are in agreement with previous descriptions of soluble αS multimers (and tetramers might be the most abundant species among them).^52–60^ Membrane-associated αS multimers, however, have also been described, e.g., in the context of mediating SNARE complex assembly^61^ and vesicle clustering.^62^ Figure 2d illustrates a model of dynamic cellular αS behavior in health, centered on the idea of membrane-assisted, transient αS helical folding and resultant multimer formation.^45^ Research from our department and other labs suggests that perturbations of a complex equilibrium between monomers and multimers, as well as between free and membrane-bound states, may represent an initial biochemical change that eventually leads to αS- and membrane-rich cytoplasmic inclusions (LBs/neurites) and progressive neurotoxicity.^59,63,64^

## αS cytotoxicity: excess membrane binding vs. fibrillar aggregation

**Fig. 3:**
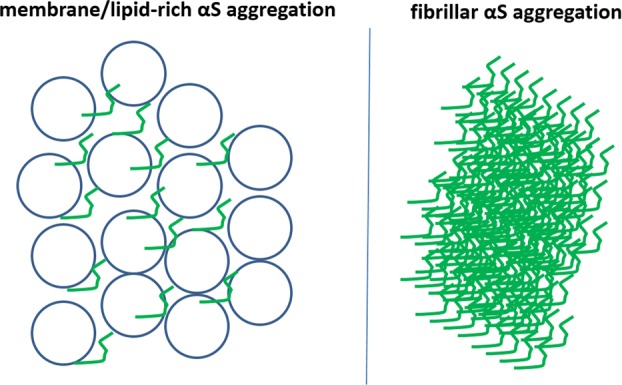
Contrasting αS fibrillar (“amyloid”) and (vesicle) membrane/lipid-rich aggregation forms. They are not mutually exclusive. Membranous aggregates could, e.g., be precursors of fibrillar aggregates.

The structure of αS suggests that transient αS–membrane interactions could be stabilized biochemically by either amplifying the electrostatic interaction between positive lysines in αS and negative lipid head groups (e.g., via the engineered “3K” αS mutant) or by increasing the hydrophobicity in the lower, membrane-inserted half of the αS amphipathic helix (e.g., via the engineered “KLK” αS mutant) (Fig. 2e). We have found that such membrane-enriched mutants of αS decrease multimers and lead to acute toxicity and inclusion formation when expressed in cultured cells. The resulting inclusions, however, were shown by electron microscopy (EM) not to be overtly fibrillar but rather rich in αS-decorated vesicles.^12^ This experimental finding was seemingly at odds with the original isolation of filamentous aggregates from LBs that were αS-positive by immunogold electron microscopy.^65,66^ However, it may be consistent with an experimental study of random αS point mutants that assessed their fibrillization in vitro (test tubes) and in living yeast.^11^ Here, in vitro fibrillization rate and in vivo yeast toxicity did not correlate, suggesting that fibrillization is not necessary for αS-induced yeast toxicity. A second screen in a library of several thousand αS-mutant yeast clones identified 25 non-cytotoxic αS sequence variants.^11^ Most of these sequence variants contained an αS mutation to either proline (P) or glutamic acid (E), which abnormally decreased αS membrane binding relative to wild-type (wt) αS. The authors hypothesized that αS cytotoxicity in yeast is caused by the protein binding to membranes at levels sufficient to nonspecifically disrupt membrane homeostasis. Subsequent yeast studies helped further characterize this membrane-associated toxicity: wt human αS expression in yeast (which lack an αS gene) led to vesicle clustering/aggregation^67,68^ and vesicle-trafficking defects.^69^ “Amyloid” was typically not obvious (see also review by Jarosz and Khurana^70^), even though at least one study also reported fibrillar aggregates upon αS expression in yeast.^71^ The relevance of the vesicle-related observations beyond yeast was supported when similar trafficking defects were described in αS A53T and αS triplication iPSC-derived human neurons.^72^ Nonetheless, the putative lack of amyloid-type αS aggregates in yeast was viewed critically by some investigators, because filaments of αS had long been considered the hallmark of human Lewy cytopathology, calling into question the relevance of the αS yeast model. A debate thus arose between those that only accepted amyloid-type αS fibrillar aggregation as disease relevant and others who were open to other forms of αS misfolding, including membrane-associated aggregation (Fig. 3). Although the former group could generally rely on the support of neuropathologists, the literature on human LBs has actually provided some evidence of membrane-associated αS aggregation.

## Membrane-associated αS aggregation in Lewy pathology

**Fig. 4:**
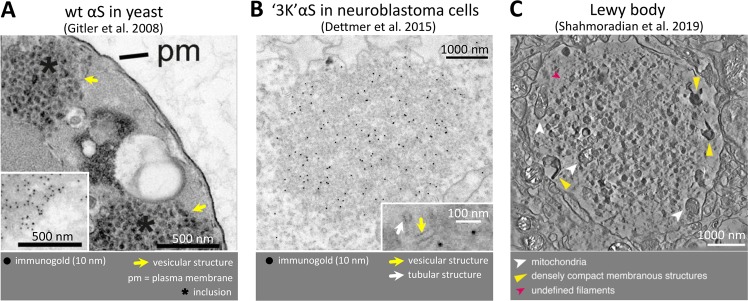
αS membrane-associated aggregation in LBs and models thereof. **a** Vesicle-rich membranous αS aggregation in the αS::GFP expression model in yeast. Insert: immunogold staining for αS. Reprinted by permission from the National Academy of Sciences, USA^68^. **b** Vesicle- and tubule-rich membranous αS aggregation in the αS 3K neuroblastoma cell model. Reprinted from Dettmer et al.^12^ (CC-BY license). **c** Vesicle/membrane/lipid-rich αS aggregation in human Lewy bodies. Reprinted by permission from the Springer Publishing Group.^13^

Early descriptions of human Lewy cytopathology in the 1960s and 70s reported filament-rich regions in LBs but also some vesicle/membrane components^73,74^ and occasional reports in the 1980s and 90s confirmed this observation.^75–77^ Nonetheless, the acceptance of this insight and its impact on conceptualizing PD pathogenesis and developing PD biomarkers and drugs has been limited so far. This situation may change after a striking recent publication: a detailed analysis^13^ of PD brain tissue by correlative light and electron microscopy, a technique that allows immunohistological and EM ultrastructural analysis of the same lesions. The authors found that the majority of human LBs consisted of αS intermingled with clusters of various membranous structures or components (Fig. 4, right). Of special significance, the authors identified in the core of LBs various vesicle clusters that were coated with high local concentrations of non-fibrillar αS. Surprisingly, only about 20% of all LBs/LNs had large amyloid fibrils (at least 5 nm diameter, at least 25 nm in length; smaller structures were likely not detectable with the method) associated with them, indicating that amyloid-type αS aggregation is not as integral or required a part of PD cytopathology as formerly believed. Raman scattering and infrared spectroscopy showed that the LB core comprised large amounts of lipids, most importantly sphingomyelin and PC, as determined by mass spectrometry. These striking new insights raise the question of why these features of human LBs were apparently overlooked in the past. As the authors point out, the short postmortem intervals of their cases and the special tissue preservation methods they applied may be responsible. Traditional LB analyses (e.g., see ref. ^78^) have largely relied on immunohistochemical staining at the light microscopic level, providing limited resolution.^79^ Thus, a bias might have been created in the past for areas that showed the expected rod-like or fibrous structures (see a commentary by Bartels^79^). The important new findings of Shahmoradian et al.^13^ are based on state-of-the-art imaging techniques and, if confirmed by others, have the potential to change the ways in which we conceptualize and model PD pathogenesis and design therapeutics.

## Membrane-associated aggregation in cellular models of αS dyshomeostasis

The proposed lipid vesicle-rich clusters within human LBs are reminiscent of the effects of expressing human αS at relatively high concentrations in *Saccharomyces cerevisiae* (see above). The αS inclusions in yeast had initially been interpreted in light micrographs as proteinaceous aggregates. Gitler et al.,^68^ however, provided ultrastructural evidence that αS accumulations in yeast were not comprised of fibrils but rather were clusters of many vesicles (Fig. 4, left), and this was confirmed by another publication.^67^ Similarly, it was observed in yeast that accumulation of undocked vesicles coalesce into massive vesicle clusters in an αS dose-dependent manner.^68^ By immunofluoresence microscopy and immuno-EM, these non-filamentous αS inclusions in yeast were associated with vesicle markers of diverse subcellular origin (endosomes, Golgi, lysosomes). In contrast to yeast, mammalian neural cells expressing very high levels of wt, or even familial PD (fPD) single-mutant αS (e.g., E46K), may not readily show discrete αS inclusion formation (e.g., see ref. ^59^). In an HEK293 model, aggregation propensity of αS was shown to be exacerbated by fPD mutants A30P, A53T, and E46K.^80^ fPD mutants A53T, A30P, E46K, H50Q, and G51D were found to have the same oligomerization propensity but differing inclusion formation in a similar HEK293 model.^81^ The exact nature of these aggregates/inclusions as well as a possible cell-type dependence of the observed inclusions will require further analysis. It is important to mention that the known fPD-linked aS point mutations have diverging effects on membrane binding: A30P binds membranes less, E46K binds more strongly, and this may indicate that the initial pathways leading to toxicity may differ between mutants (see review^50^ for further details). Indeed, the degree to which the protein binds membranes in mammalian models seems to be recapitulated in yeast models of αS toxicity, e.g., αS A53T was shown to localize to (plasma) membranes, whereas A30P remained cytoplasmic, recapitulating the poor membrane binding nature of A30P reported in many models.^82^ To further elucidate the relationship between αS membrane binding and aggregation/toxicity, engineered αS mutations may prove to be useful tools. Certain engineered mutations in the αS KTKEGV motifs that decrease the cellular multimer:monomer ratio increase the interactions of the excess αS monomers with membranes Such mutations include αS “3K” (E35K + E46K + E61K),^59^ an amplification of the familial PD-causing E46K (Fig. 2e) or “KLK” (T12L + T23L + T34L + T45L + T60L + T71L + T82L; Fig. 2e).^64^ These exaggerated, membrane-enriched mutations have provided evidence that the vesicle-clustering property of human αS is not unique to yeast but can also be achieved in the time course of αS expression in cultured mammalian neural cells (Fig. 4, middle). In this context, it is important to note that the trafficking and clustering of membrane vesicles within synaptic terminals has been suggested to be a normal function of αS (reviewed in ref.; ^83^ see also a recent review on the physiological role of αS and its relationship with PD^84^). This raises the possibility that the abnormal vesicle clustering seen in αS-expressing yeast and in the vesicle-accumulating KTKEGV motif mutants represent a form of excessive αS function (a “toxic gain-of-function”). Interestingly, isolated monomeric, but not multimeric, αS was shown to interact with membranes in the test tube (presumably via helix formation, as the folding-deficient A30P did not interact), leading to membrane remodeling and tubulation.^55^ Intact-cell crosslinking of neurons expressing inclusion-forming αS KTKEGV variants such as “3K” and “KLK” (Fig. 2e) suggests that αS in the membrane-rich inclusions may be principally monomeric. This raises the question of the nature of αS molecules in the vesicle-rich inclusions: are they amphipathic helices, the species that was shown to form at vesicles? Are they unfolded? Or are they early-stage β-sheet oligomers undetectable by crosslinking or yellow fluorescent protein (YFP) complementation? The first scenario, which we favor, would be most provocative, because the field of protein misfolding diseases is used to the dichotomy “helical fold = good, β-sheet = bad”.

## Is there a bidirectional interplay between αS and lipids?

**Fig. 5:**
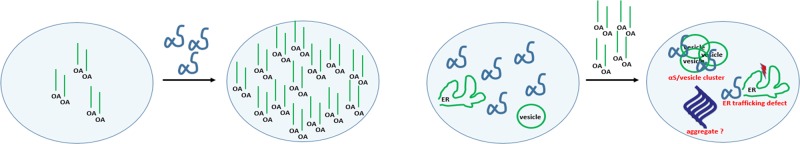
Indications for an αS ↔ OA bidirectional pathogenic loop. αS excess leads to increased OA levels; increases in OA disrupt αS homeostasis leading to αS + vesicle clusters, vesicle-trafficking defects, and possibly αS aggregates. Inhibition of the rate-limiting enzyme in OA production, (SCD), promises to mitigate these pathogenic events.

In addition to altering vesicle trafficking, expressing αS in yeast was shown to promote lipid droplet (LD) formation.^82,85^ Changes in LD content and distribution have been associated with αS toxicity and membrane-trafficking defects in yeast and mammals (reviewed in ref. ^86^). Wt αS in mammalian cells has been proposed to bind to LDs^85^ and this binding propensity may be amplified by certain natural (E46K) or engineered (3K) KTKEGV mutants, which accumulate on membranes; the resultant cytoplasmic vesicle aggregates are often in the vicinity of LDs (see Figs 2 and 3 in ref. ^12^). Moreover, it was recently reported that αS expression in yeast and αS excess in rodent neurons or induced pluripotent stem cell (iPSC)-derived human neurons lead to marked alterations in lipid profiles, including increases in neutral lipids.^14^ Among FAs, oleic acid (OA) was found to be specifically elevated in response to excess αS monomers.^14^ Strikingly, reciprocal effects were also seen in this^14^ and a related^16^ study: lowering the enzymatic formation of monounsaturated FAs (MUFAs; e.g., OA) appeared to benefit αS biochemistry; it increased physiological αS multimerization, increased αS solubility, and decreased serine 129 phosphorylation. In contrast, conditioning cells with MUFAs had the opposite effects. These observations align in part with earlier work, demonstrating pathological αS oligomer accumulation upon conditioning cultured neural cells with polyunsaturated FAs (PUFAs).^7^ The proposed αS-OA interplay is consistent with a scenario in which excess αS, in particular membrane-associated αS monomers, leads to an increase in MUFA levels, which in turn render αS more neurotoxic (Fig. 5). Such a model is reminiscent of a “bidirectional pathogenic loop” that had been proposed for another cellular lipid, glucocerebrosidase (GCase), and αS^87^ (see below). A novel therapeutic strategy emerging from the work just summarized as follows: inhibiting the rate-limiting enzyme in the biosynthesis of MUFAs, stearoyl-CoA desaturase (SCD). This approach, which emerged simultaneously from another group based on unbiased compound screens in αS-expressing yeast,^15^ could potentially (a) neutralize the upregulation of MUFAs by excess αS and (b) prevent detrimental structural changes in αS that are the consequence.

## Human genetics, patient samples, and experimental models link PD to lipid pathways

The theory we emphasize here that lipid metabolism is central to αS homeostasis is particularly well supported by human genetic evidence that strongly suggest a key αS-lipid interplay (Fig. 1) and a major role for certain lipids in modulating αS physiology and consequent toxicity in the brain.^11,88^ This concept is in line with a systematic analysis of GWAS data and genetic networks that revealed lipid homeostasis as a common link between several processes involved in PD pathogenesis.^89^ GWAS have identified—and postmortem brain analyses have confirmed—several proteins that help regulate lipid metabolism, including LD biology, to be associated with PD. First and foremost, mutations in *GBA* (GCase), a key gene in glycolipid metabolism, significantly increase PD risk^20,21^—and certain other genes in related pathways have also been implicated^26,27^ (see below). In addition, a diacylglycerol kinase, *DGKQ*, which controls diglyceride and phosphatidic acid content, emerged from several GWAS as a PD risk factor.^17,22–25^ FA elongase 7, a determinant of fatty acyl side-chain length, and hence membrane composition and fluidity, was recently designated as another significant PD risk gene.^28,90^ A phospholipase, *PLA2G6*, has been proposed to affect risk for PD and other brain diseases with “high brain iron”^91^. Furthermore, seipin, an integral membrane protein localized at endoplasmic reticulum (ER)/LD contact sites and involved in LD biogenesis and maintenance,^92,93^ may be differentially expressed in the brains of PD vs. control subjects.^94,95^ These findings suggest phospholipid group and side-chain nature (dictated by FA type) play a critical role in PD, likely through αS interaction alterations. As far as non-cell-autonomous lipid homeostasis is concerned, variants in LRP10, a low-density lipoprotein receptor protein, were also reported be linked to PD dementia, DLB, and Lewy pathology,^96,97^ but this remains controversial^98^ (LRP10 mutations have also been associated with increased Alzheimer’s disease pathogenesis^99^). Moreover, *SREBF-1*, a transcription factor that binds sterol regulatory element-1 and controls lipid homeostasis through sterol biosynthesis, has been identified in GWAS as a PD risk factor.^26^ This finding, in addition to certain studies on statin use, suggests that sterol pathways in PD pathogenesis should not be ignored either.

We hypothesize that the downstream effects of PD-relevant lipid alterations may involve changes in vesicle trafficking and vesicle function (lysosomes and synaptic vesicles in particular). Several known and emerging PD risk genes such as *LRRK2* (*PARK8*), *RAB7* (*PARK16*), *VPS35* (*PARK17*), *SYNJ1* (*PARK20*), *VPS13C* (*PARK23*), *SYT11*, and *LIMP2* (all reviewed in ref. ^86^) underline the relevance of vesicle trafficking and function in PD pathogenesis, and both are affected by lipid and/or αS alterations (further details are beyond the scope of this review). Focusing on putative upstream events and in the context of a possible “bidirectional interplay” between lipids and αS, the following paragraphs will summarize in some detail what genetics, patient samples, and model systems have taught us about how certain lipid species may alter αS biology—and how they might be altered by αS.

## Glycolipids

Although only slightly increasing disease risk (unlike *SNCA* and *LRRK2*), *GBA* (GCase) mutations are the most common risk factor for PD. Homozygous *GBA* mutations block proper sphingomyelin metabolism and cause Gaucher’s disease, a developmental disorder characterized by lysosomal dysfunction. Heterozygous mutations (i.e., in Gaucher’s carriers) have repeatedly been found to increase PD risk.^20,21^ A lack of GCase and the resultant glucosylceramide build-up promotes increased formation of abnormal oligomers of αS. In turn, elevated levels of these neurotoxic αS species result in reduced lysosomal GCase activity, which further stabilizes αS oligomers. Increasing lysosomal GCase, thereby decreasing αS oligomer formation, disrupts this pathogenic loop.^87^ By way of follow-up, the same group found that increasing lysosomal GCase activity in iPSC-derived dopamine neurons from patients with PD-associated *GBA* mutations reduced αS accumulation.^100^ In keeping with this concept, a study of the impact of *GBA* deficiency on αS homeostasis found that glycosphingolipid accumulation resulting from *GBA* loss-of-function decreased physiological αS multimers and increased the more aggregation-prone monomeric form. In accord, transfecting in wt *GBA* or applying the drug miglustat (which blocks a synthetic enzyme for glycosphingolipids) restored the physiological αS multimer:monomer ratio and decreased cytotoxicity.^101^ In mice with age-dependent reductions in GCase function, aberrant lipid association of αS (and tau) was found in a subset of Secretogranin II + large dense-core vesicles responsible for neurotransmission of dopamine and other monoamines.^102^ In addition to *GBA*, an association between mutations in the sphingomyelin phosphodiesterase *SMPD1* or in *ASAH1*, a lysosomal ceramidase, and PD were identified recently.^27^ Moreover, deficiency in *GALC*, a lysosomal enzyme involved in the catabolism of galactosylceramide, may contribute to neuronal vulnerability in late-onset synucleinopathies.^103^

## Phosphatidylcholine

PC is the most abundant phospholipid in cellular membranes.^104^ Decreased levels of PC containing the polyunsaturated fatty acyl side chains denoted 34:5, 36:5, and 38:5 were observed in the frontal cortex of PD brains.^105^ Similarly, PD visual cortex has been reported to have reductions in some PC species with polyunsaturated 34 and 36 carbon species, as well as decreases in 16:0, 18:0, 18:1, and 18:2 lyso-phosphatidylcholines (LPCs).^106^ In a study of PD patient plasma, PC 44:6 and 44:5 were increased and PC 35:6 was decreased.^107^ Such changes in PUFAs could be the consequence of αS accumulation on membranes, because trends for lower PC species were observed in yeast and rat cortical neuron models of αS excess.^14^ Treating rats with the dopaminergic neurotoxin 6-hydroxydopamine^108^ led to early (defined by the authors as preceding “full blown primary symptoms”) lipid changes in the substantia nigra (SN), with most PC species decreased. Exceptions were LPC 16:0 and LPC 18:1, which were increased. Interestingly, sex differences in lipid changes in PD patients have been observed: in one study, PC was significantly decreased in male patients only.^109^ An in vitro study, highlighting the importance of using native αS forms for studies, reported that PC affects conformation and aggregation of the *N*-acetylated form of αS, specifically that *N*-acetylation enhances binding to PC micelles and small unilamellar vesicles with high curvature.^110^

## Phosphatidylethanolamine

Phosphatidylethanolamine (PE) is the next most abundant phospholipid after PC, comprising ~25% of total mammalian cellular phospholipids.^111^ In the brain ~45% of phospholipids are PE.^112^ Using magnetic resonance spectroscopic imaging, decreased PE (and PC) were observed in brains of early (Hoehn and Yahr stages I and II) PD patients but not in advanced (Hoehn and Yahr stages III and IV) cases.^113^ Decreases in multiple PE species (PE 34:2, 34:1, 36:4, 36:3, 36:1, 38:7, 38:6, 38:4, 40:6, 40:5, 40:4, 38p:7, and 40p:7) and Lysophosphatidylethanolamine species (16:0, 18:0, and 20:0p), particularly in the visual cortex, of PD patients relative to controls were reported.^106^ PE 36:3 was reduced in the amygdala and species 34:1, 36:3, and 36:2 were decreased in the anterior cortex cingulate of PD patients.^106^ PE 34:1 was found to be decreased in PD patient plasma.^112,113^ A sex difference, as observed for PC above, was also noted for PE, with significantly lower PE levels in male PD patients (vs. controls) but not in females.^109^ The consequences of reduced PE for αS phenotypes have been analyzed by genetically reducing cellular PE content. Deletion of a phosphotidylserine (PS) decarboxylase (*PSD1*), which synthesizes PE from PS, increased cytoplasmic αS inclusion formation and enhanced αS toxicity in a yeast model. Importantly, dopaminergic neuron degeneration from expressing wt human αS was enhanced by PSD1 RNAi silencing in a *Caenorhabditis elegans* model of synucleinopathy, whereas supplementation with ethanolamine, a building block for PE synthesis, led to partial rescue.^114^ By way of follow-up, low levels of PE in the mitochondria resulted in ER stress and induced αS to form cytoplasmic foci in this model. Feeding with ethanolamine rescued this phenotype.^115^ Trends for lower levels of shorter chain PE species were observed in yeast expressing wt human αS, whereas longer-chained PE increased. Alterations in PE species appeared αS dose- and time-dependent in a rat cortical neuron model of αS accumulation.^14^

## Phosphatidylinositol

Phosphatidylinositol (PI) is the third or fourth most abundant phospholipid in cells (after PC, PE, and potentially PS).^104^ A study in rat brain identified PI 18:0/20:4 as the major PI species and stated that all major PI species contain at least one PUFA.^116^ PI decreases with age in both male and female mice, whereas lyso-PI decreases in females only.^117^ The PI total lipid class was shown to be significantly reduced in the SN of male PD patients relative to controls.^109^ Decreased PI, particularly saturated PI species, was observed in yeast expressing human αS, whereas rat and human cortical neuron models overexpressing αS trended to decreased PI also.^14^ Although not the most prominent phospholipid or lyso-phospholipid, changes in the visual cortex and amygdala included decreases in PI 38:5 and increases in PI 36:1, 38:3, and 40:4 in PD vs. control brain.^106^

## Phosphotidylserine

Phosphotidylserine (PS) is a quantitatively minor membrane phospholipid that makes up 2–10% of total phospholipids in mammalian cells.^112^ PS is an important precursor of mitochondrial PE, which is produced by the mitochondrial enzyme PS decarboxylase. As part of a study analyzing membrane phospholipid synthesis of the SN in PD vs. control brains, it was observed that such synthesis may increase during the course of PD development,^118^ suggesting increased PS, PE, and/or PC would be observed in PD patients. In this regard, increases in PD frontal cortex of specific PS species have been observed, namely PS with 36:1, 36:2, and 38:3 fatty acyl side chains. PS has also been suggested to play a role in regulating αS-facilitated synaptic vesicle docking by aiding SNARE complex formation.^119^ Lipid raft levels of PS were 36% higher in the brains of “incidental PD” patients (cases with brain stem LB pathology but devoid of motor symptoms), but not in typical PD brains, relative to controls.^120^

## Conclusions: proteinopathy vs. lipidopathy in PD and related brain diseases

PD has been principally thought of heretofore as a classical “proteinopathy”—a disease that is caused by the misfolding of a protein into β-sheet-rich fibrillar aggregates. In this scenario, neurons are typically thought to suffer from an imbalance between protein synthesis or folding and protein degradation, leading gradually to neuronal dysfunction and death. Both reduced^63^ and excess^10^ αS–membrane interactions, as well as interactions with certain FAs^7^ have been discussed as potential triggers for toxic αS oligomerization and fibril formation, consistent with the concept of a “lipid-induced proteinopathy”. However, recent advances in LB analysis and PD modeling now provide evidence for the opposite sequence, i.e., synucleinopathies may be “protein-induced lipidopathies”. In this hypothesis, an imbalance in cellular lipid homeostasis is the actual neurotoxic process and αS dyshomeostasis (e.g., excess or reduced vesicle binding of αS associated with fibrillar aggregation) is the trigger. Perhaps it is more sensible not to propose a dichotomy: PD and related human synucleinopathies may simultaneously be proteinopathies and lipidopathies, and a vicious cycle of dyshomeostasis in protein folding and lipid metabolism might be triggered by early and subtle changes in either lipid or protein handling (the initial alteration may differ from case to case).

Although support for all three scenarios can be found in previous and recent research, the question becomes whether it is important to put a conceptual label on the disease or more scientifically relevant to keep both protein and lipid alterations in mind when exploring new ideas for therapeutics. As an example, the inhibition of SCD, a new therapeutic target, may indeed both counteract changes in lipid metabolism that are triggered by αS accumulation and prevent any negative feedback of these lipid changes on αS structure.^14^ Regulation of FAs other than MUFAs may also prove valuable, given the report that arachidonic acid (20:4) promotes the formation of helical αS multimers, and these may resist fibril formation and result in reduced neuronal damage.^60^

The emerging evidence that αS accumulation in LBs may not solely be fibrillar as traditionally assumed has several important implications. First, the popular “PFF” model of αS fibrillization and proteotoxicity^121,122^ may have to be rethought and complemented with models of intracellular membrane-mediated αS aggregation such as the αS “3K” model that was developed in cultured cells^59^ and then shown in transgenic mice to cause PD-like phenotypes.^123^ Second, positron emission topography tracers for synucleinopathies may have to be designed differently than attempted thus far, taking into account the protein’s excess on cellular membranes in a yet undefined conformation. Third, drugs that alter lipid and FA homeostasis in addition to SCD inhibitors may become promising targets.^14–16^ Fourth, the common assumption that proteinopathy means amyloid aggregation may have to be modified in the case of αS in PD and DLB. From these and other considerations, a new model is emerging in which αS “misfolding” in the cell can occur on several levels. Just an excess amount of the wt αS monomer that normally interacts with vesicles, i.e., which forms physiological amphipathic helices, may already have to be considered a type of “misfolding”. Thus, excess membrane-associated αS may be cytotoxic and disease relevant in the absence of actual β-sheet-rich fibrillar aggregation. A progression to β-sheet-rich fibrillar aggregates may confer further detriment, but it might also be temporarily beneficial by sequestering αS monomers away from membrane vesicles, thereby mitigating negative effects on vesicle trafficking. The recently published αS “3K” mouse model that exhibits a pronounced neurodegenerative and movement disorder phenotype indeed developed vesicle-rich αS + aggregation in young animals, whereas older animals seemed to display occasional “classical” filament-rich amyloid aggregates.^123^ This finding could indicate that vesicle clustering is potentially an early event in LB formation, whereas fibrillar aggregates are characteristic of more mature inclusions. Related to these considerations may be the question of the nature of the so-called “pale bodies” of αS aggregation that can be observed in PD patient brains and that have been discussed to potentially be a stage in the formation of LBs (e.g., see ref. ^124^).

It should also be noted that the genetics of αS mutations per se suggest that two ways of LB formation may exist (recently reviewed in ref. ^50^): one via excess membrane binding of monomers (E46K, A53T) and one via excess accumulation of soluble monomers in the cytosol (A30P, G51D). Both pathways seem to have in common the accumulation of monomers at the expense of putative physiological multimers.^59^

**Fig. 6:**
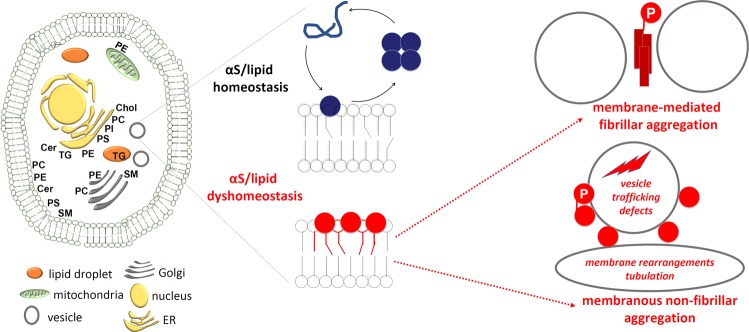
αS/lipid homeostasis and dyshomeostasis. Left: cell with LDs, vesicles, ER, nucleus, mitochondria and annotated with some of the lipid species discussed in this review and relevant to PD. Parts are adapted from Van Meer et al.^104^ Cer ceramide, Chol cholesterol, PC phosphotidylcholine, PE phosphotidylethanolamine, PI phosphatidylinositol, PS phosphotidylserine, SM sphingomyelin, TG triglyceride. Middle: intact αS/lipid homeostasis (top: αS transiently binds to membranes and undergoes cycles of assembly and disassembly) and αS lipid dyshomeostasis (bottom: αS accumulates at membranes of altered composition). Right: αS in disequilibrium forms fibrillar (top) or membranous non-fibrillar aggregates, mediated by abnormal lipid interactions.

Our final figure (Fig. 6) summarizes the potential pathways leading to membrane-associated and fibrillar αS aggregation we have emphasized in this review; the bidirectional aspects of the interplay are highlighted. In the final analysis, lipid homeostasis appears to loom ever larger in the fundamental mechanisms of human synucleinopathies.
